# Terahertz waves dynamic diffusion in 3D printed structures

**DOI:** 10.1038/s41598-022-12617-3

**Published:** 2022-05-21

**Authors:** Mauro Missori, Laura Pilozzi, Claudio Conti

**Affiliations:** 1 Institute for Complex Systems, National Research Council, Via dei Taurini 19, 00185 Rome, Italy; 2 Department of Physics, University Sapienza, Piazzale Aldo Moro 5, 00185 Rome, Italy; 3 Research Center Enrico Fermi, Via Panisperna 89a, 00184 Rome, Italy

**Keywords:** Photonic crystals, Optical materials and structures, Metamaterials

## Abstract

Applications of metamaterials in the realization of efficient devices in the terahertz band have recently been considered to achieve wave deflection, focusing, amplitude manipulation and dynamical modulation. Terahertz metamaterials offer practical advantages since their structures have typical sizes of hundreds microns and are within the reach of current three-dimensional (3D) printing technologies. Here, we propose terahertz photonic structures composed of dielectric rods layers made of acrylonitrile styrene acrylate realized by low-cost, rapid, and versatile fused deposition modeling 3D-printing. Terahertz time-domain spectroscopy is employed for the experimental study of their spectral and dynamic response. Measured spectra are interpreted by using simulations performed by an analytical exact solution of the Maxwell equations for a general incidence geometry, by a field expansion as a sum over reciprocal lattice vectors. Results show that the structures possess specific spectral forbidden bands of the incident THz radiation depending on their optical and geometrical parameters. We also find evidence of disorder in the 3D printed structure resulting in the closure of the forbidden bands at frequencies above 0.3 THz. The size disorder of the structures is quantified by studying the dynamics diffusion of THz pulses as a function of the numbers of layers of dielectric rods. Comparison with simulations of light diffusion in photonic crystals with increasing disorder allows estimating the size distributions of elements. By using a Mean Squared Displacement model, from the broadening of the pulses’ widths it is also possible to estimate the diffusion coefficient of the terahertz radiation in the photonic structures.

## Introduction

Diffraction of light by corrugated surfaces is a key optical phenomenon studied since the early days of the electromagnetic theory^1–9^. It is exploited in many applications^10^ including extremely high-Q filters^11^, ultra-broadband reflectors^12^, wavelength selective polarizers^13^ and beam splitters^14^, to name a few. In the past few years, diffraction of light inspired the design of artificial interfaces by spatially arranging subwavelength planar microstructures (e.g. “meta-atoms”) as fundamental building blocks. Such metasurfaces allow manipulating light on a subwavelength regime, changing properties such as phase, amplitude and polarization. They have been recently exploited to realize interesting effects in the field of flat optics^15,16^, topological photonics^17^ and generation of geometrical phases^18,19^. Recently, the focus has shifted to vertical stacking of patterned layers^20^ where the interlayer distance and the layers number may act as a new degree of freedom to modify the optical band structure. The subject is presently far from being exhausted.

Specifically, potential applications of metasurfaces in the realization of efficient devices in the terahertz (THz) band (i.e., 0.1–30 THz) have recently been considered^21^ ranging from wavefront shaping^22^ to manipulation of circularly polarized THz beams^23^. Both passive and active THz structures have been considered to achieve wave deflection^24^, focusing^25^, amplitude manipulation^26^ and dynamic terahertz waves modulation^27^. THz radiation has a fundamental relevance^28^ in a variety of fields ranging from astronomy to biology and it is crucial for the development of information technologies, including sixth-generation (6G) communication and THz integrated circuits. This growth of important applications in diverse fields calls for the development of devices with new functionalities and all-dielectric metamaterials have emerged as a promising platform for highly efficient THz systems^29–32^.

THz metamaterials offer practical advantages, namely, their structures have typical sizes of hundreds microns and they are within the reach of current three-dimensional (3D) printing technologies^33^. In general, this technology is able to create 3D objects through additive manufacturing (AM) methods, where the final product is obtained layer by layer. AM concepts have been applied in a multitude of ways, bringing a wide range of 3D printing technologies allowing cheap, rapid and customisable fabrication of THz components made of polymers, ceramics, metals, and composites^34,35^.

Among the different AM technique the fused deposition modeling (FDM) is considered as the most commonly available, widely used, and cost effective for various applications. In FDM, a thermoplastic filament is melt and fed through to a preheated nozzle to the build platform in a predetermined pattern. Once the material gets cooled down and solidified to form a layer, the build platform moves in the vertical direction and the nozzle dispenses another layer on top of the previous layer subsequently. This process is repeated, until the whole object is complete. Thanks to the continuous innovation of hardware, materials, and processes, structures with minimum features sizes and tolerances around 100–200 $\upmu$m can be fabricated by using FDM, providing a low-cost solution for rapid manufacturing of THz components working in the lower frequency region of the THz band^34–36^.

However, size tolerance values are not completely negligible with respect to the wavelength of low frequency THz radiation and therefore the build structures are affected by an inevitable presence of intrinsic disorder due to size or position dispersion of the building blocks occurring during the growth process. Over a threshold of the scatterers polydispersivity, the onset of a diffusion process for light propagation can be reached that may eliminate many functionalities of subwavelength photonic devices^37^. Scattering due to intrinsic or controlled disorder may eventually lead to effects like Anderson localization^38^, random lasing^39,40^, light focusing^41^.

In this work we report on an experimental study of THz pulses propagation in the diffusive regime to investigate the effects and quantify the amount of disorder in FDM multilayer structures made of free-standing arrays of rectangular rods of dielectric polymeric material. Previous works on the same topic were focused on single grating: the angular dependence of the diffracted beams of a 3D printed single grating were compared to theoretical simulations^42^, and Mie resonances were characterized by using THz time-domain spectroscopy (THz-TDS) in a dielectric grating obtained by direct-laser-writing technique on a Si wafer and compared to finite-element simulations^29^. We also experimentally characterize the 3D printed photonic structures by measuring the transmittance of the multilayer stacking by THz-TDS. This technique allows experiments where the temporal shape of ultra-short THz pulses transmitted through a sample can be measured^43^. We employ this approach to study the THz pulses dynamics as well as their spectral behaviour by converting the THz time signals into spectral data.

**Figure 1:**
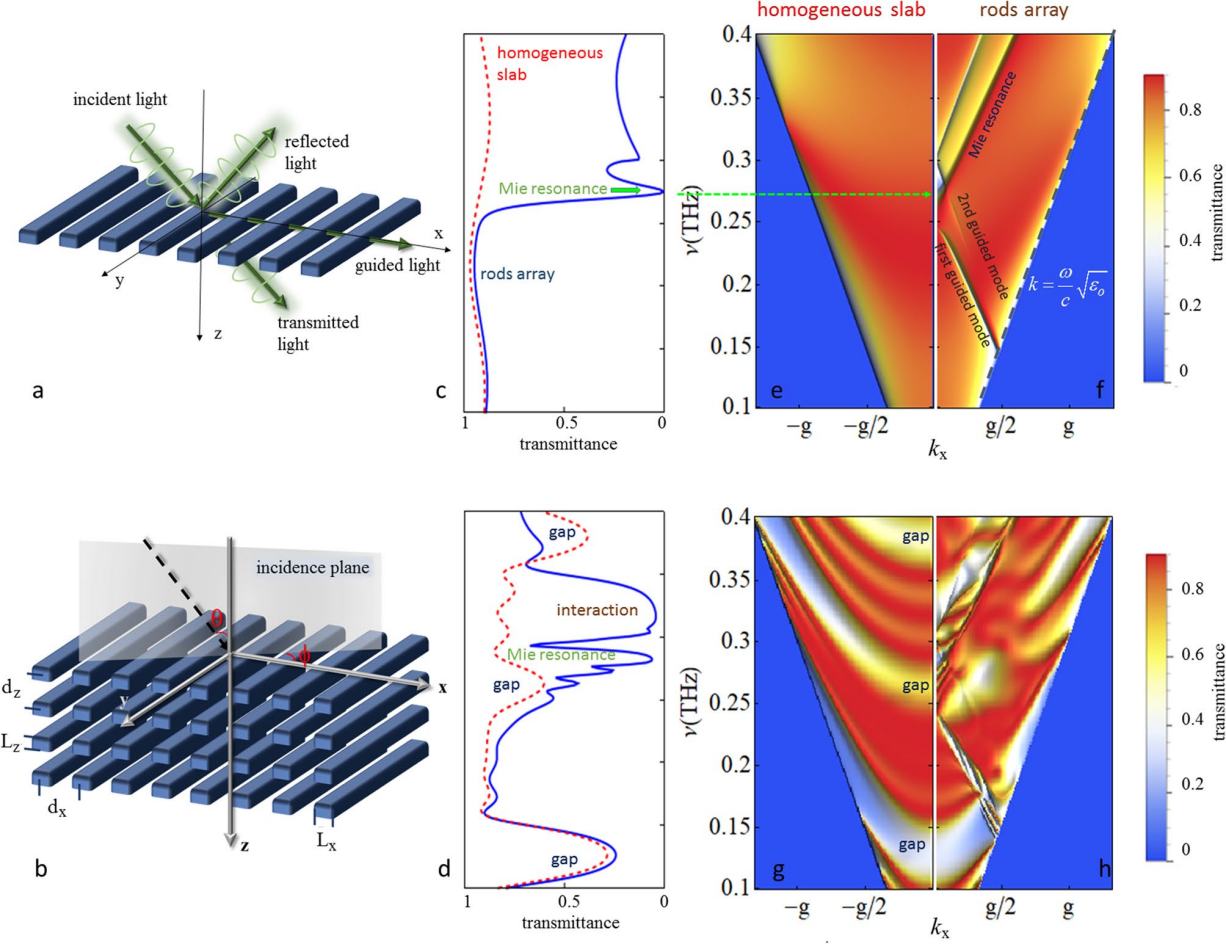
(**a**) Schematic representation of the single layer of dielectric rods and (**b**) the multilayer structure; (**c**) theoretical transmittance at normal incidence for the homogeneous slab (red dashed curve) and the rods array (blue solid line) for the single layer and (**d**) 4 layers of rods (the frequency values $\nu$ are the same of vertical axes of maps (**e,g**); (**e**) transmittance map as a function of frequency and $k_x$ for the single homogenous layer, and **f** for the single layer of rods, (**g**) four homogeneous slabs and (**h**) four layers of rods. All the plots are for $\phi =0$, $L_x=550\, \upmu$m, $d_x=1000\, \upmu$ m, $L_z=495 \,\upmu$m and $d_z=990 \,\upmu$m.

In order to design the structures and interpret the experimental results we provide a theoretical description of electromagnetic radiation propagation by solving Fourier transformed Maxwell equations for the single layer and then applying boundary conditions to analyze the vertically stacked structure. This method allows to study systems with a finite thickness and obtain their optical properties as a function of the number of layers. Despite being a very simple structure, a single layer of rods array may be designed to show interesting properties as broad reflection bands with rather high reflectivity and large scalability^44^ and negative trasmission where the trasmitted wave at the first diffracted order has the maximum intensity^12^.

The fabrication method exploited in our study allows to investigate the different modes of the multilayer stack: guided and Mie resonances^45,46^ as well as bound states by shaping the single layer and Bragg gaps formation for an increasing layer number. However the size randomness in the structure causes the closure of the forbidden bands by tails of states at the band edges. As a main result, the dynamics of the THz pulses through the multilayer system allows to estimate its intrinsic randomness and then the quality of the printed structures. Indeed, exponential light localization due to disorder induces a diffusive regime of light propagation^47,48^ with a consequent slowing down of photons, resulting in the development of an exponential temporal tail or an increasing width of the THz pulses of the propagating beam. Therefore, measuring the width of trasmitted THz pulses enable us to quantify the diffusion constant of the photonic structures.

The method used in this work moreover allows to describe the hybrid scattering regime that arises in realistic periodic optical structure where usually both order and disorder play a role. Due to the unavoidable imperfections light undergoes multiple scattering which can be described as a diffusion process. At the same time, order may inhibit propagation, giving rise to photonic band gaps, resulting in a vanishing diffusion constant, due to the formation of localized eigenstates. In our analysis as a function of the layer’s number, we manage to quantify the degree of disorder in the single layer. The same method can also be used to observe the onset of the hybrid scattering regime in photonic structures.

## Results

### Structure design

Our structures, as shown in Fig. 1 are one-dimensional (1D) periodic arrangements of self-sustained rods (Fig. 1a), as THz metasurfaces, and their multilayer composition (Fig. 1b), as THz photonic crystal slabs. The single layer periodicity is along the x direction, the rods axis is along the y direction while the multilayer structure is obtained by staking the single layer along z. The incidence plane, normal to the rods axis, forms an angle $\phi$ with the x axis, while $\theta$ gives the orientation of the incident wave with respect to the z axis. To design them we, first, observe that each layer acts, at the same time, as a diffraction grating and an in-plane slab waveguide, with the resulting formation of guided mode resonances. The single layer geometric parameters, periodicity $d_x$, rods width $L_x$ and thickness $L_z$, and the interlayer distance, defining the period $d_z$, make the multilayer structure to show, for a given dielectric function $\varepsilon _b(\omega )$, specific dispersion bands and spectral features for the transmitted THz radiation. Indeed, defining the light line, $\omega _l=ck_p$ with *c* the speed of light in vacuum and $\user2{\vec{k}}_p=k_x\hat{x}+k_y\hat{y}$ the in-plane wavector, for the single layer we can have resonances or bound modes for frequencies above or below the light line, and forbidden bands formation with increased layer number $n_\ell$.

These characteristics can be easily identified in the spectra and transmittance maps shown in Fig. 1c–f. The transmission spectrum for normal incidence condition ($\theta =\phi =0$) of the single layer (blue curve in Fig. 1c) shows the characteristic Mie resonance, where nearly-perfect optical extinction occurs^49^, at a frequency value connected with the ratio $L_z/L_x$ of the elementary rod. The spectrum exactly match the transmittance curve of an homogeneous slab (red dashed curve in Fig. 1c) of dielectric function $\overline{\varepsilon } =f_f\,\varepsilon _b(\omega ) + (1 - f_f)\varepsilon _o$, defined by the grating filling factor $f_f=L_x/d_x$, in the frequency range till the onset of the second traveling wave in the grating.

The same features are present in the maps of Fig. 1e,f for the single layer and g,h for the photonic slab, where the transmittance amplitude for $\phi =0$ is given as a function of the frequency and the in-plane wave-vector $k_x$, i.e. the incidence angle. The single homogeneous layer (Fig. 1e), $L_z=495\, \upmu$m thick and with a $f_f = 0.55$, shows the usual Fabry–Perot oscillations. The map for the single free-standing grating (Fig. 1f) shows minima in the transmittance amplitudes due to the Mie resonance and the folded guided modes. Their dispersions show couplings that lead either to band gap opening or crossings, depending on their strength. Worth noting is that, at normal incidence, additional modes can be present. Indeed, since our photonic crystal slab possess a 180$^\circ$ rotational symmetry about the z-axis (C2), symmetry-protected bound states in the continuum (BICs)^50^ with frequency above the light line, may exist. They are not related to some form of disorder in the lattice but the confinement of these radiationless states is due to the symmetry mismatch between the folded mode and the Bloch modes of the structure^51^.

Increasing the number of layers in the stack, additional features are present. Specifically, for $n_\ell =4$, as shown in Fig. 1g,h, Bragg gaps open for both the homogeneous and the rods system. In addition, the interplay among folded modes and Mie resonances is responsible for the features in the map of Fig. 1h, in particular for the gap at about 0.32 THz.

**Figure 2:**
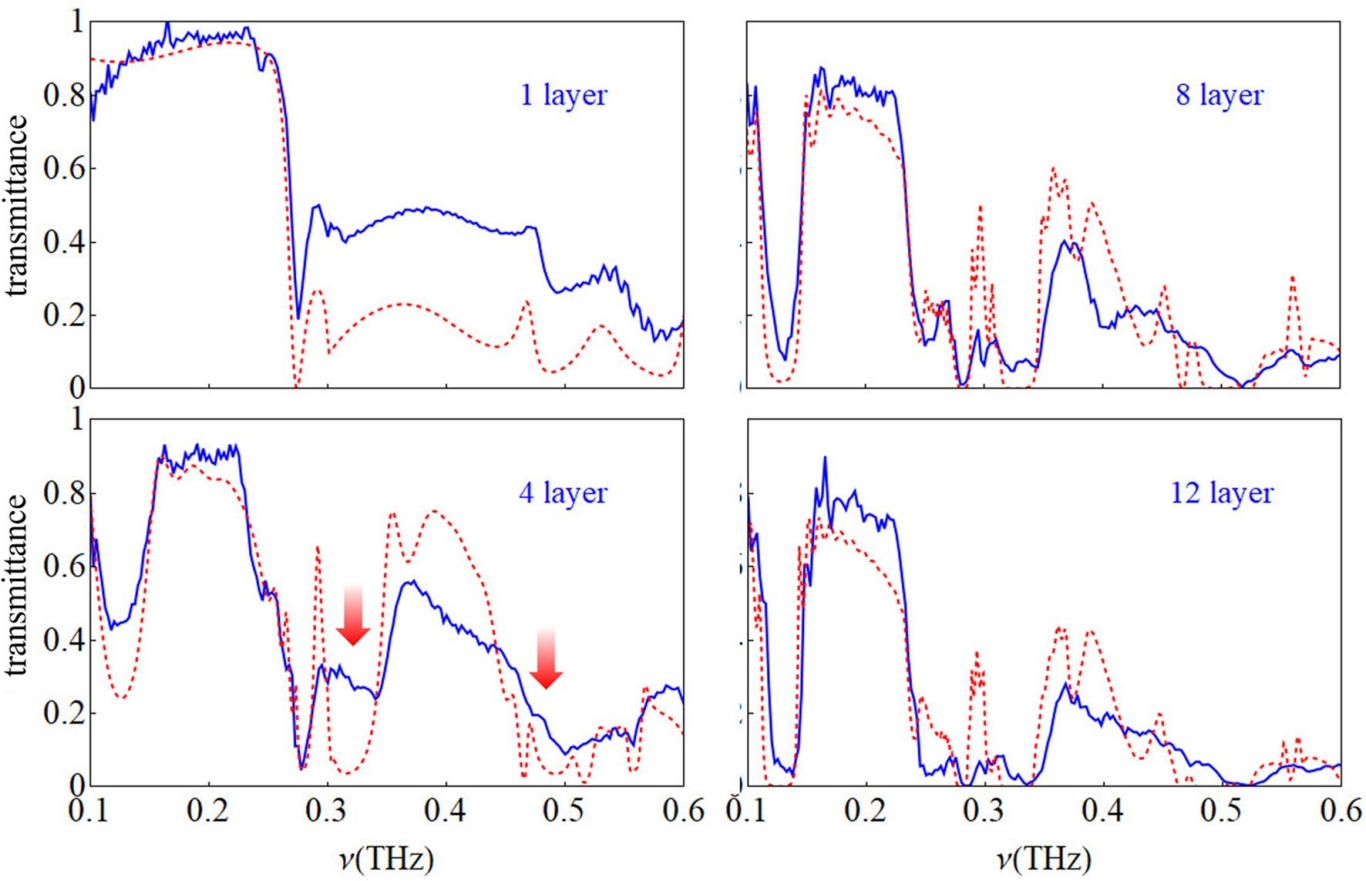
Comparison between experimental and simulated transmittance spectra for photonic structures made of 1, 4, 8 and 12 layers of rods. For 1 layer the agreement is very good with only an overall higher transmittance for the experimental curve after 0.3 THz. For 4, 8 and 12 layers the agreement is still good overall but differences between the experiments and simulations appear after 0.3 THz. In particular, the most evident difference is the closure of the forbidden bands which can be well seen in the structure with 4 layers (red arrows).

All the plots in Fig. 1 correspond to the transmittance of the zero order diffraction. This condition corresponds to transmission along the THz beam line axis and these spectra can therefore directly be compared to the measured ones. A comparison between experiments and simulations from 0.1 to 0.6 THz are shown in Fig. 2 for structures made of 1, 4, 8 and 12 layers of rods. For one layer the agreement is qualitatively very good. Quantitatively, an overall higher transmittance for the experimental curve is observed for frequencies higher than the Mie resonance, which shows a minimum at 0.275 THz, and may be likely due to tapered sidewall profiles^52^.

For 4 layers of rods the first Bragg gap appears at about 0.13 THz. The Mie gap appears at 0.278 THz, a value slightly different from that of one layer of rods, indicating some randomness in the structure. The gap simulated at about 0.32 THz result to be poorly defined in the measured spectrum. In general for 4 and 8 layers of rods the agreement is good overall below 0.3 THz, but more evident differences between experiments and simulations appear after that frequency value. For 12 layers of rods the differences are more pronounced.

In general, the most evident difference between experiments and simulations for 4, 8 and 12 layers of rods is the closure of the forbidden band gaps. This is a well known effect in photonic crystals and it is due to the increasing disorder in the structure: the photonic band gap is closed by tails of states at the band edges. It was demonstrated that a few percent of randomness is sufficient to close the forbidden bands^53^. Disorder effects in the structures can be expected due to the non negligible values of the size tolerances of FDM with respect to the THz wavelength. Deviations between experiment and theory with increasing frequencies were also observed in a 3D printed Wollaston prism and attributed to the size tolerance of the printing process^54^.

### THz pulses dynamic diffusion

**Figure 3:**
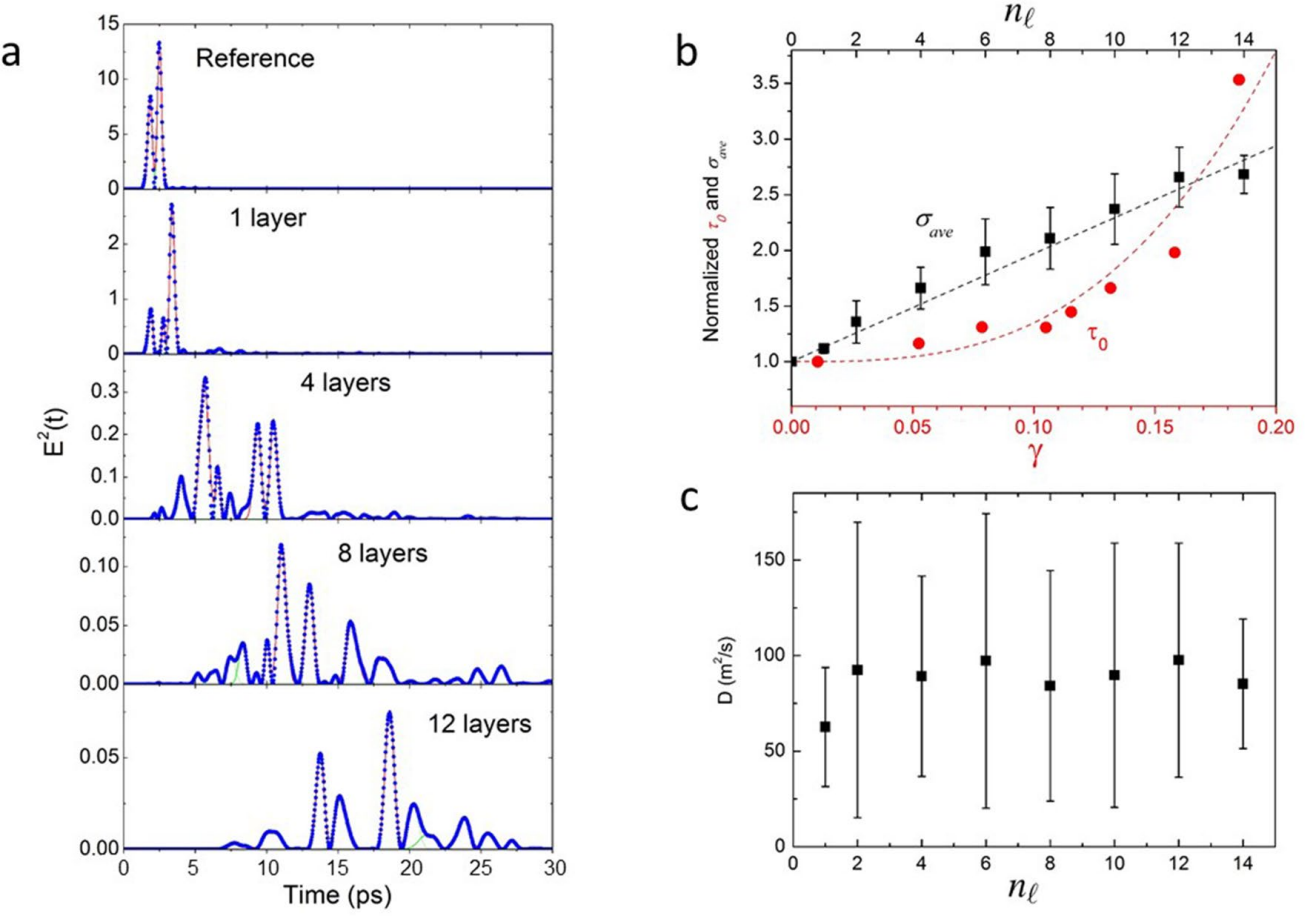
(**a**) The squared module of transmitted THz pulses as a function of $n_\ell$. (**b**) The normalized THz pulses $\sigma _{ave}$ as a function of $n_\ell$ (top x axis) and the graph of the normalized $\tau _0$ as a function $\gamma$ (bottom x axis). The black dashed line is the linear fit of $\sigma _{ave}$, the red dashed curve is a third-degree polynomial fit of $\tau _0$. (**c**) The diffusion constant of the THz pulses within the photonic structures as a function of $n_\ell$ showing a constant behaviour.

**Figure 4:**
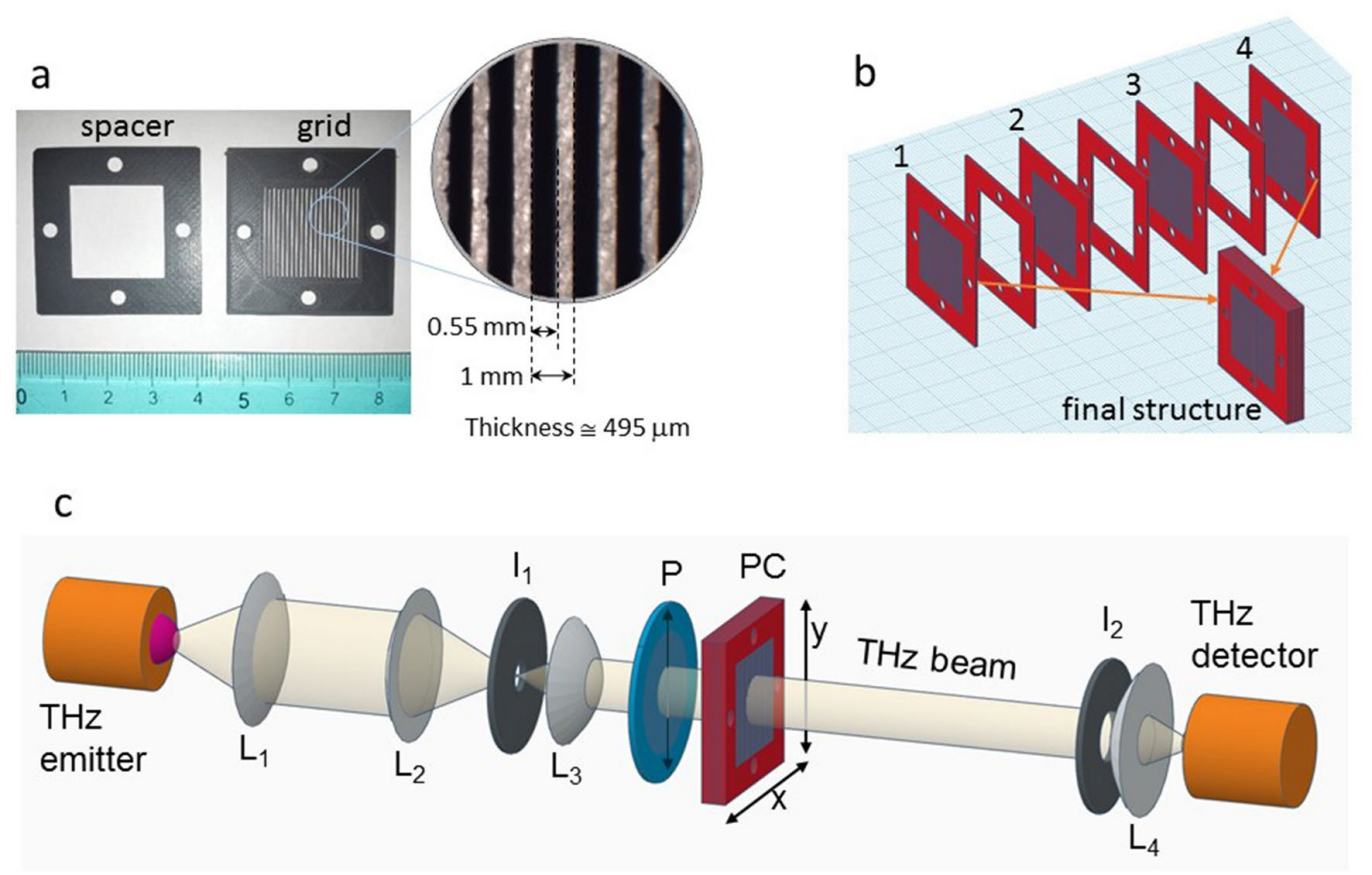
(**a**) The spacer and the grid, the 3D printed elements of the fabricated multilayer structures with their mean sizes. (**b**) The assembly of grids and spacers to build a structure made of a square lattice of rods with 4 layers. Other structures were assembled with 1 to 14 layers of rods. (**c**) Sketch of the THz beam line used for the experiments (see main text for details).

In order to provide a quantitative evaluation of the disorder in the photonic structures, we investigate the dynamic diffusion process of the incident THz pulses. This study is possible because the employed THz-TDS spectrometer allows measuring the shape of the ultrashort THz pulses transmitted through the structures with a resolution of 33.3 fs^55^. First, we transformed the THz signal, which is proportional to the electric field, in its squared module. The squared module of the input THz pulse signal is made up of two pulses (Fig. 3a, reference graph). As a function of the numbers of layers, the number of trasmitted pulses and their time delay increases (Fig. 3a, 1, 4, 8 and 12 layers graphs). In addition, an overall increasing of the pulses’ width is observed as a function of the number of layers of rods. For instance, the standard deviation $\sigma$ obtained from a gaussian best fit of the main pulses of the reference and that transmitted by the 12 layers’ structure increases from 0.148 to 0.397 ps, respectively.

This last effect is interpreted as due to the critical slowing down of the photons in disordered structures^53,56^. In order to obtain a quantitative description of this phenomena the average of $\sigma$, $\sigma _{ave}$, of all pulses with intensity greater than 10$\%$ of the maximum pulse transmitted by each structure is calculated. The values of $\sigma _{ave}$ normalized to that of the reference pulses are shown in Fig. 3b (black filled squares) as a function of the $n_\ell$ (top scale), where the error bar is the standard deviation of $\sigma _{ave}$ for each structure. The behaviour of $\sigma _{ave}$ can be well fitted by a linear function $1+0.193 n_\ell$ (black dashed line). Interestingly, a similar behaviour of the pulse width can be obtained by a deconvolution procedure applied to the time-dependent data as described in Ref.^56^. Results of this procedure are shown in the Supplementary information.

The comparison with simulations of the trailing edge of optical pulses in disordered inverted opals^53^ allows us to estimate the disorder of the 3D printed structures. These simulations provide the decay time constant $\tau _0$ of the intensity of an ultrashort optical pulse as a function of the structural fluctuations where the radius *r* of each element of the inverted opal is given by $r=r_0(1+\gamma \xi )$, where $r_0$ is the radius of each sphere in the absence of disorder, $\gamma$ is the degree of disorder and $\xi$ is a uniform deviate in $[-\,1/2, 1/2]$ ($\gamma =0$ is the ordered case).

$\tau _0$ is calculated for an inverse opal photonic crystal of fixed size with increasing size disorder of its spherical elements, using a parallel Finite-Difference Time-Domain (FDTD) proprietary software code^53^. The 3D printed photonic structures, instead, have an increasing thickness depending on $n_\ell$ but reasonably similar size and position disorder of their elements (rods) due to the non negligible tolerances of FDM technology with respect to THz wavelength, in particular for frequencies greater than 0.3 THz (wavelength 1 mm).

The plot of $\tau _0$ normalized to that obtained without disorder as a function of $\gamma$ (Fig. 3b, red circles, bottom scale) increases in a monotonic way and can be fitted by a third order polynomial $1+350 \gamma ^3$ (Fig. 3b, red dashed line).

We can recover the value of $\gamma$ associated to a single layer of rods by equating the increment of the normalized $\sigma _{ave}$ due to the addition of a single layer, 0.193, to the increment due to the degree of disorder $350 \gamma ^3$. Solving gives $\gamma =0.082$.

This value well agree with the measured dispersion of $L_x$ of about 0.07, $L_z$ of about 0.05, and $d_x$ of about 0.08, obtained by microscope images analysis of the grids used in the photonic structures (see “Photonic crystals fabrication” section). These results demonstrate the validity of the comparison of THz pulse width with the simulated $\tau _0$.

The small values of $\gamma$ indicate that the photonic structure is a small scattering system. In highly scattering systems a scale-dependent diffusion coefficient is needed to explain anomalies in the temporal shape of pulses of light transmitted through a sample. In our case we estimate the diffusion coefficient, *D*, of the THz radiation in the photonic structures from the spreading of the pulses’ widths by using a Mean Squared Displacement (MSD) model. This is used to describe transport processes in many materials from the broadening of the gaussian profile of a perturbation^57^, where:

1 $$\begin{aligned} D = \frac{[\sigma _{ave}^2(n_\ell )-\sigma _{ave}^2(0)]c'^2}{2t}, \end{aligned}$$

where $\sigma _{ave}^2(n_\ell )$ is the average gaussian variance of the temporal profile of the THz pulses as a function of $n_\ell$, $\sigma _{ave}^2(0)$ is that of the reference pulses and *t* is the time the pulses need to pass through each photonic structure. $c'$ is the speed of the THz radiation in the photonic structures and it is calculated as:

2 $$\begin{aligned} c'=c \frac{f_z+n_\ell -1}{n_\ell f_z\overline{n}+(n_\ell -1)(1-f_z)n_o}, \end{aligned}$$

where $f_z=L_z/d_z$, *c* is the speed of the light in vacuum and the mean refractive index of the equivalent slab model of a layer of rod is calculated as $\overline{n} =f_f\, n _b(\omega ) + (1 - f_f) n_o$, with $n_b$ and $n_o$ the refractive index of rods and air, respectively. $\overline{n}=1.33$ for $f_f=0.55$, $n_b=1.60$ and $n_o=1$. *t* is calculated as the sum of the propagation times in the grid layers and in the spacers of the photonic structures:

3 $$\begin{aligned} t= \frac{n_\ell L_z \overline{n}}{c} + \frac{(n_\ell -1)( d_z-L_z)n_o}{c}= \frac{d_z}{c} \bigg [n_\ell \overline{n}f_z+(n_\ell -1)n_o(1-f_z)\bigg ]. \end{aligned}$$

Results for *D* as a function of $n_\ell$ are shown in Fig. 3c: *D* appears to have constant values of ($90\pm 20$) m$^2$/s when varying $n_\ell$ even if it shows a large error mainly due to the uncertainty on $\sigma _{ave}^2(n_\ell )$.

## Discussion

By using the FDM 3D printing technique multilayer structures of rods layers (from 1 to 14 layers) were successfully fabricated. Simulations of their THz optical response were performed by an analytical exact solution of the Maxwell equations resulting in good agreement with transmittance experimental spectra, in particular for THz frequencies below 0.3 THz. Simulations allowed interpreting the observed spectral behaviour as Mie, Bragg and interaction bands.

Differences between experiments and simulations appear for multilayers structure with $n_\ell \ge 4$ for frequencies above 0.3 THz. The most evident difference is the closure of the forbidden band gaps which is interpreted as due to the increasing disorder in the structure. Disorder effects in the 3D printed structures can be expected due to the non negligible values of the size tolerances of FDM (100–200 $\upmu$m) with respect to the THz wavelength. Indeed, it was demonstrated that a few per cent of randomness is sufficient to close the forbidden bands in photonic crystals.

In order to provide a quantitative evaluation of the disorder in the structures, we investigate the dynamic diffusion process of the incident THz pulses exploiting the temporal resolution of THz-TDS. We based our investigation on simulations of $\tau _0$ of optical pulses in disordered inverted opals as a function of $\gamma$. We recover $\sigma _{ave}$ by performing a gaussian best fit of the transmitted pulses, finding that it can be fitted by a linear function as a function of $n_\ell$. $\tau _0$ increases monotonically and can be fitted by a third-degree polinomial as a function of $\gamma$. The comparison of the behavior of $\sigma _{ave}$ and that of $\tau _0$ provide an estimation of $\gamma$ of a single layer of rods ($\gamma =0.082$). This provide a first quantitative information on the randomness of the structure based on the THz dynamic diffusion. The calculated value of $\gamma$ is in agreement with measurements of size and period dispersion of the rods, which range from 0.05 to 0.08.

In addition, by employing a Mean Squared Displacement (MSD) model to describe transport processes of THz photons in the 3D printed photonic structures it is possible to estimate their diffusion constant *D*. The obtained constant value of *D* of ($90 \pm 20$) m$^2$/s as a function of $n_\ell$ appears to be reasonable. In fact, since each layer of rods is made by using the same fabrication method and, moreover, the structure was created by composing layers with rod sizes and periodicity within a specific range, it is reasonable to suppose that they have similar disorder making *D* independent of $n_\ell$.

These results demonstrate the feasibility of the 3D printed multilayer structures for the applications in the THz frequency band. The estimation of the size disorder allow to interpret their THz spectral behavior as due to the competition of band gap formation due to order and their closing because of the disorder.

The broadening of the pulses may not only result from the disorder of the photonic structures but also on the frequency dependence of the absorption coefficient and refractive index of ABS as well as on the dispersive character of each single layer of rods (grating), which can give even pulse temporal compression. Since the overall pulse behaviour is complex for a photonic crystal of rods, thorough numerical simulation are needed for a detailed analysis: in this manuscript our aim is to present the experimental data and extract a quantitative parameter such as the diffusion constant for future comparison with numerical simulations.

This characterization open the way to the fabrication of complex 3D printed photonic structures for THz range with specific optical responses. An example could be the implementation of twisted multilayer structures where the relative in-plane rotation of the single planes can act as a new geometrical degree of freedom to engineer the photonic bands, in analogy with twisted bilayer 2D materials^58^.

## Methods

### Photonic crystals fabrication

The photonic crystals (PC) were fabricated in Acrylonitrile Styrene Acrylate (ABS) by using an Anycubic i3 Mega (China) 3D printer using the FDM technology with 0.4 mm diameter nozzle. In order to have a better control of the sizes of printed components separate elements were printed to realize the PC. The printed elements are layers of rectangular rods (from now on also called grid) and spacers (Fig. 4).

After 3D printing each element was measured in order to recover the grid period and the rectangular rod width by using size calibrated images obtained by using a stereo microscope STMPRO-T BEL Engineering (Italy) equipped with a EUREKAM 3.0 3MPixel camera. BEL View 7 software was used for image acquisition and ImageJ for measurements of grids features. The same analysis showed slightly tapered sidewall profiles of the rods. To compose the photonic crystal elements with grid period $d_x=(1000 \pm 40)$ $\upmu$m and rectangular rod width $L_x=(550 \pm 20)$ $\upmu$m were sorted. The thickness of grids and spacers was measured by using a mechanical caliper with 1 $\upmu$m sensitivity: grids and spacers with an average thickness $L_z=(495 \pm 12)$ $\mu$m were sorted for the realization of the photonic crystals. The grids were arranged together to the spacer in order to built photonic crystals made of from 1 to 14 layers of rods (Fig. 4b). In addition, ABS slabs with a thickness of 0.5 and 2 mm were 3D printed to measure the THz optical parameters of the ABS used for the photonic structures.

### Measurement setup

The spectral and dynamical properties of THz pulses transmitted through the photonic crystals were measured by using a TERA K15 THz-TDS (Menlo Systems, Germany). This set-up is equipped with a photoconductive antenna excited by a femtosecond fiber coupled laser (Menlo Systems T-Light). The laser emission wavelength was 1560 nm, the repetition frequency 100 MHz, and the pulse duration approximately 90 fs. THz time-domain data were acquired with a time resolution of 33.3 fs. The THz beamline was realized to have a gaussian beam with size smaller than that of the PCs (Fig. 4c). Radiation emitted by the photoconductive antenna was collected and focused into an iris (I$_1$) by means of two TPX (polymethylpentene) lenses (L$_1$ and L$_2$) with 50 mm nominal focal length. The diverging THz radiation after I$_1$ was collected and focused by a 25 mm nominal focal length HRFZ-Si lens (L$_3$) in a beam of about 15 mm diameter. Before impinging on the PC THz radiation passed through a wire polarizer (P), model PW010-030-050 from Pure Wave Polarizers (UK), oriented to have the electric field vector of THz radiation parallel to the rods. THz radiation transmitted by samples passed through another iris (I$_2$) open to the same beam size and focused on the detector by a second 25 mm nominal focal length HRFZ-Si lens (L$_4$).

For all acquisitions the delay line scan range was 400 ps (the resulting spectral resolution is therefore 2.5 GHz), and the scan rate was about 2 Hz. For each acquisition the THz signals were averaged over 109 scans (each lasting about 0.46 s), therefore the time for each sample or reference acquisition was 50 s. THz-TDS signals were converted into their spectral representation by a fast Fourier transform (FFT). The usable spectral range was found to be 0.1–3.0 THz. The frequency dependent dynamic range was about 70 dB at 0.35 THz. The PCs were mounted in a bidirectional translation stage allowing movement in a xy plane orthogonal to the THz beam. The transmission spectra were averaged on 9 different positions within the PC each separated by 1 mm along the x and y axes.

The same set-up was used to measure the dielectric function of the ABS material as a function of frequency. To this purpose transmittance of 3D printed unstructured ABS slabs with thickness of 0.5 and 2 mm were measured. The optical parameters were obtained from the measured spectra by using a numerical procedure able to remove the residual Fabry–Perot oscillations present in the spectral behaviors of samples with flat and parallel surface^59^. The obtained values of the real $\varepsilon _1$ and imaginary $\varepsilon _2$ dielectric functions as a function of frequency were fitted by a linear function $a+b \omega$ (*a* and *b* are 2.566 and $-\,0.0814$ for $\varepsilon _1$ and 0.0315 and 0.0520 for $\varepsilon _2$) and then used in the simulations. These values are in agreement with previous measurements of ABS optical parameters^35^. Graphs of the used ABS absorption coefficient, refractive index and dielectric functions as a function of frequency are shown in the Supplementary information.

### Theory

Each layer in our system is a free-standing 1D array of rectangular rods of period $d_x$, with dielectric function $\varepsilon _b(\omega )$ (in vacuum $\varepsilon _o$), with translational symmetry along the x-axis, as shown in Fig. 1a. The dimensions of each rod are $\underline{L} _x$ and $\underline{L} _z$ and the grating filling factor is $f_f = \underline{L} _x/d_x$, so that an average weighted dielectric function, that defines an equivalent slab model of the grating can be given as $\overline{\varepsilon } =f_f\varepsilon _b(\omega ) + \left[ {1 - f_f} \right] \varepsilon _o$.

We describe the grating dielectric tensor:

4 $$\begin{aligned} \varepsilon (x,\omega ) = \varepsilon _o + F(x)\left[ {\varepsilon _b(\omega ) - \varepsilon _o } \right] , \end{aligned}$$

through the sum of products of two Heaviside functions:

5 $$\begin{aligned} F (x) = \mathop {\lim }\limits _{N \rightarrow \infty } \sum \limits _{\ell = - N}^{ N}{\vartheta [{x - (x_\ell - L_x/2)}}]\vartheta [{(x_\ell + L_x/2) - x}], \end{aligned}$$

where $x_\ell = \ell \;d_x$ is the x coordinate of the center of the $\ell{\text{-th}}$ rod with $\ell =0, \pm \, 1, \pm \, 2,....\pm \, N$.

The Fourier transformed dielectric tensor is then:

6 $$\begin{aligned} \varepsilon (G,G') = \varepsilon _o \delta _{G,G'}+ \frac{L_x}{d_x}\left[ \varepsilon _b(\omega ) - \varepsilon _o \right] \frac{sin(\alpha _{GG'})}{\alpha _{GG'}}, \end{aligned}$$

where $G=m2\pi /d_x=mg$ is a reciprocal lattice vector of the grating and $\alpha _{GG'}=(G - G')L_x/2$. Its diagonal elements, i.e. the limit $G\rightarrow G'$, describe the equivalent slab while the off-diagonal ones give rise to band splitting and to a folding of the photonic modes in the first Brillouin zone. Following Ref.^60^ we solve Fourier-transformed Maxwell’s equations as a generalized eigenvalue problem. Reflectivity and transmittance are then obtained by imposing Maxwell’s boundary conditions at the planes delimiting the grating layers. This approach, exploiting plane wave expansion, allows to handle multilayer photonic crystal slabs where standard tools for the theoretical studies of photonic crystals, relying upon periodicity features, cannot be directly applied^61^.

For a general incidence condition, we expand the electric field components ($\alpha = x, y, z$), in the j-th grating layer, in plane waves:

7 $$\begin{aligned} E_{j\alpha }(\rho ,z,\omega )=e^{ikz}\sum _{G}E_{j\alpha }(\vec {q}_p+G\hat{x},k,\omega )e^{i(\vec {q}_p+G\hat{x})\rho }, \end{aligned}$$

where $\rho =(x,y,0)$ and $\vec {q}_p=q_x\hat{x}+q_y\hat{y}$ is the in-plane wavevector. This allows to reduce Maxwell equations to a generalized eigenvalue problem of the form^12^:

8 $$\begin{aligned} \mathbf{A} \varvec{\eta }&= k^2\mathbf{B} \varvec{\eta }. \end{aligned}$$

The details of this derivation are given in the Supplementary Information.

In Eq. (8), the matrices **A** and **B** are partitioned into four (2N + 1) $\times$ (2N + 1) blocks. The diagonal blocks $\mathbf{A} _{ii}$ and $\mathbf{B} _{ii}$ refer to TE (i = x) and TM (i = y) polarizations while the off-diagonal ones introduce polarization mixing. In the particular case where the incidence plane is orthogonal to the rods, i.e. $\phi = 0$ and $q_y=0$, the off-diagonal blocks of both **A** and **B** are null and $\mathbf{B} _{yy}$ reduces to the identity matrix. Consequently the system in (8) separates in two eigenvalue problems for two independent, TE and TM, polarizations^60^. Moreover, given N the value to ensure the numerical convergence of the calculation, one ends with 2N + 1 square roots $k_n^2$ of the eigenvalue problem, and 2N + 1 eigenfunctions $\eta _n(G)$ of 2N + 1 components.

Finally, by writing the electric field in each layer region $L_z$ thick, in the form:

9 $$\begin{aligned} E_{j\alpha }(\rho ,z,\omega )=\sum _{n}\left(\chi ^>_{nj}e^{ik_nz}+\chi ^<_{nj}e^{-ik_nz}\right)\sum _{G}E_{j\alpha }(\vec {q}_p+G\hat{x},k_n,\omega )e^{i(\vec {q}_p+G\hat{x})\rho }. \end{aligned}$$

The application of Maxwell boundary conditions, at the interfaces of the multilayer system, allows to obtain the amplitudes of the forward $\chi ^>_{nj}$ and backward $\chi ^<_{nj}$ propagating waves.

These conditions require the continuity of the in-plane component of the electric field and the normal component of the displacement field and have to be set up, for each Fourier component (G), on the two surfaces delimiting each grating layer. (2N + 1) $\times$ (2N + 1) diagonal phase matrices of elements $e^{\pm iq_z(G)(d_z-L_z)}$ allow to match the fields in consecutive grating layers. The ± sign refers to the forward (+) and backward (−) propagating waves.

We cast the problem, of defining the fields amplitudes, in the scattering matrix form to handle possible numerical instabilities due to the presence of evanescent waves for high reciprocal-lattice vectors^62^.

The full description of how the applications of Maxwell boundary conditions allow to describe the multi-layer structure in the scattering matrix formalism is given in the Supplementary information section.

## Supplementary Information

Supplementary Information.
