# Supplementary material for: Terahertz waves dynamic diffusion in 3D printed structures
Source: Sci Rep. 2022 May 21;12:8613. doi: 10.1038/s41598-022-12617-3 (PMC9124215; doi:10.1038/s41598-022-12617-3)
Supplement: Supplementary file 1 — Supplementary Information. [file 41598_2022_12617_MOESM1_ESM.pdf]

Supplementary informations
Terahertz waves dynamic diffusion in 3D printed structures

Mauro Missori[1,2], Laura Pilozzi[1,3,*], and Claudio Conti[1,2,3]

[1]Institute for Complex Systems, National Research Council, Via dei Taurini 19, 00185 Rome, Italy
[2]Department of Physics, University Sapienza, Piazzale Aldo Moro 5, 00185 Rome, Italy
[3]Research Center Enrico Fermi, Via Panisperna 89a, 00184 Rome, Italy
[*]laura.pilozzi@isc.cnr.it

ABSTRACT

In this supplementary section we describe (1) the approach we adopted in reducing Maxwell equations into a generalized eigenvalue problem (Eq.(8) of the main text), how we solved it and (2) how the applications of Maxwell boundary conditions allow to describe the multi-layer structure in the scattering matrix formalism. Moreover, we provide (3) a plot of the real and imaginary part of the refractive index and the dielectric functions of the material (ABS) used for the photonic structures realization and (4) the deconvolution technique applied to the THz time-domain data.

1 Generalized eigenvalue problem.

In this section we describe our approach to solve Maxwell equations in a patterned layer, specifically a single layer of rods array. For a general incidence condition, the electric field components ($\alpha = x, y, z$), in the j-th grating layer, expanded in plane waves, read as:

$$E_{j\alpha}(\rho, z, \omega) = e^{ikz} \sum_{G} E_{j\alpha}(\vec{q}_p + G\hat{x}, k, \omega) e^{i(\vec{q}_p + G\hat{x})\rho} \tag{S1}$$

where $\rho = (x, y, 0)$ and $\vec{q}_p = q_x\hat{x} + q_y\hat{y}$ is the in-plane wavevector. In the following we omit the j index since, in the structures considered, the grating layers are all the same.
By magnetic field elimination, Maxwell equations can be written as:

$$\nabla \times \nabla \times \mathbf{E}(\mathbf{r}) = \mathbf{D}(\mathbf{r}) \tag{S2}$$

and, once Fourier transformed, in mixed coordinates, they turn into:

$$\begin{cases} \left[\frac{\partial^2}{\partial z^2} - q_y^2\right] E_x(G,z) + q_x(G) q_y E_y(G,z) - i q_x(G) \frac{\partial E_z(G,z)}{\partial z} = -\frac{\omega^2}{c^2} D_x(G,z) \\ \left[\frac{\partial^2}{\partial z^2} - q_x^2\right] E_y(G,z) + q_x(G) q_y E_x(G,z) - i q_y(G) \frac{\partial E_z(G,z)}{\partial z} = -\frac{\omega^2}{c^2} D_y(G,z) \\ i q_x(G) \frac{\partial E_x(G,z)}{\partial z} + i q_y \frac{\partial E_y(G,z)}{\partial z} + \left[q_x^2(G) + q_y^2\right] E_z(G,z) = \frac{\omega^2}{c^2} D_z(G,z) \end{cases} \tag{S3}$$

where: $q_x(G) = q_x + G$. Moreover, given the dielectric displacement field in the form:

$$D_\alpha(G,z) = \sum_{G'} \varepsilon_{G,G'} E_\alpha(G',z) = e^{ikz} \sum_{G'} \varepsilon_{G,G'} E_\alpha(G',k) \tag{S4}$$

they reduce to

$$\begin{cases} \sum_{G'}\left[\frac{\omega^2}{c^2}\varepsilon_{G,G'} - q_y^2\delta_{G,G'}\right]E_x(G',k) + q_x(G)q_yE_y(G,k) = k^2E_x(G,k) - kq_x(G)E_z(G,k) \\ \sum_{G'}\left[\frac{\omega^2}{c^2}\varepsilon_{G,G'} - q_x^2\delta_{G,G'}\right]E_y(G',k) + q_x(G)q_yE_x(G,k) = k^2E_y(G,k) - kq_yE_z(G,k) \\ \sum_{G'}\left[\frac{\omega^2}{c^2}\varepsilon_{G,G'} - (q_x^2(G)+q_y^2)\delta_{G,G'}\right]E_z(G',k) = -k\left[q_x(G)E_x(G,k) + q_yE_y(G,k)\right] \end{cases} \tag{S5}$$

The former system can be solved with respect to the z component of the field:

$$E_z(G,k) = -k\sum_{G'}\mathbf{M}^{-1}_{G,G'}\left\{q_x(G')E_x(G',k) + q_yE_y(G',k)\right\} \tag{S6}$$

with the matrix elements $M_{G,G'} = \frac{\omega^2}{c^2}\varepsilon_{G,G'} - (q_x^2(G') + q_y^2)\delta_{G,G'}$.
The substitution of the expression in Eq.(S6) in the first two equations of the system of Eq.(S5) turns the Maxwell equations in a generalized eigenvalue problem:

$$\begin{pmatrix} \mathbf{A}_{x,x} & \mathbf{A}_{x,y} \\ \mathbf{A}^T_{x,y} & \mathbf{A}_{y,y} \end{pmatrix}\begin{pmatrix} E_x \\ E_y \end{pmatrix} = k^2\begin{pmatrix} \mathbf{B}_{x,x} & \mathbf{B}_{x,y} \\ \mathbf{B}^T_{x,y} & \mathbf{B}_{y,y} \end{pmatrix}\begin{pmatrix} E_x \\ E_y \end{pmatrix} \tag{S7}$$

where the matrix blocks, of dimension $(2N+1)\times(2N+1)$, are given by :

$$\begin{array}{l} \mathbf{A}_{x,x}(G,G') = \frac{\omega^2}{c^2}\varepsilon_{G,G'} - q_y^2\delta_{G,G'} \\ \mathbf{A}_{x,y}(G,G') = q_x^2(G')q_y\delta_{G,G'} \\ \mathbf{A}_{y,y}(G,G') = \frac{\omega^2}{c^2}\varepsilon_{G,G'} - q_x^2(G')\delta_{G,G'} \\ \mathbf{B}_{x,x}(G,G') = q_x(G)\mathbf{M}^{-1}_{G,G'}q_x(G') + \delta_{G,G'} \\ \mathbf{B}_{x,y}(G,G') = q_x(G)\mathbf{M}^{-1}_{G,G'}q_y \\ \mathbf{B}_{y,y}(G,G') = q_y\mathbf{M}^{-1}_{G,G'}q_y + \delta_{G,G'} \end{array} \tag{S8}$$

Notice that with the superscript T we indicate vector transposition.
In order to transform the system in Eq.(S7) into a canonical problem, we solve the eigenvalue system: $\mathbf{A}\alpha_n = \lambda_n\alpha_n$, with:

$$\mathbf{A} = \begin{pmatrix} \mathbf{A}_{x,x} & \mathbf{A}_{x,y} \\ \mathbf{A}^T_{x,y} & \mathbf{A}_{y,y} \end{pmatrix} \tag{S9}$$

Given $\mathbf{U}$ the unitary transformation that diagonalizes $\mathbf{A}$, namely $\mathbf{U}^T\mathbf{A}\mathbf{U} = \lambda$, by multiplying Eq.(S7) by $\mathbf{U}^T$ on the left, we obtain:

$$\lambda\tilde{\eta} = k^2\tilde{\mathbf{B}}\tilde{\eta} \tag{S10}$$

where $\tilde{\eta} = \mathbf{U}^T\eta$ and $\tilde{\mathbf{B}} = \mathbf{U}^T\mathbf{B}\mathbf{U}$, with $\eta = (E_x, E_y)^T$.

Finally, defining $\tilde{\tilde{\eta}} = \lambda^{1/2}\tilde{\eta}$ and $\tilde{\tilde{B}} = \lambda^{-1/2}\tilde{B}\lambda^{-1/2}$ we manage to reduce the generalized eigenvalue problem (Eq.S7) to a canonical form:

$$\tilde{\tilde{B}}\tilde{\tilde{\eta}} = \frac{1}{k^2}\tilde{\tilde{\eta}} \tag{S11}$$

Eventually, the electric-field components (E_x, E_z) are computed by transforming the vector $\tilde{\tilde{\eta}}$ back to the vector η.

2 Multilayer solution

In this section we describe how the multi-layer solution can be obtained starting with the simple case of a system composed of two grating layers, as shown in Fig.S1. Moreover we take the in-plane wave vector along the x axis, i.e. we consider the case

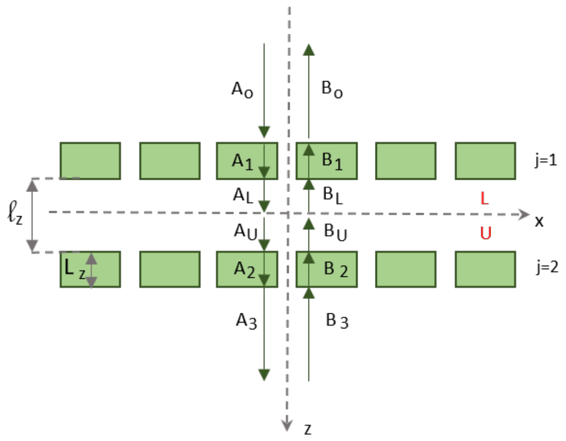

Figure S1. Schematic of the two gratings (j=1,2) configuration with indicated the forward A_i and backward B_i propagating field amplitudes in the different layers.

$\phi_o = 0$. For these conditions the in-plane electric field components in the different regions of the structure can be written as:

$$
\begin{aligned}
E_U(\rho,z,\omega) &= \sum_G e^{iq_x(G)x}\left[A_U(G)e^{iq_z(G)(z-\ell_z/2)} + B_U(G)e^{-iq_z(G)(z-\ell_z/2)}\right]\\
E_2(\rho,z,\omega) &= \sum_n \left[A_{n2}e^{ik_n(z-\ell_z/2)} + B_{n2}e^{-ik_n(z-\ell_z/2)}\right]\sum_G \eta_n(G)e^{iq_x(G)x}\\
E_3(\rho,z,\omega) &= \sum_G e^{iq_x(G)x}\left[A_3(G)e^{iq_z(G)(z-L_z-\ell_z/2)} + B_3(G)e^{-iq_z(G)(z-L_z-\ell_z/2)}\right]
\end{aligned} \tag{S12}
$$

where the k_n 2N + 1 eigenvalues and the $\eta_n(G)$ correspondent 2N + 1 eigenfunctions with 2N+1 G-components are computed for a chosen $q_x(0)$ value in the first Brillouin zone $(-\pi/d_x, \pi/d_x)$.

A phase matrix

$$
\chi(\ell_z) = \begin{pmatrix}
e^{iq_z(-N2\pi/d_x)\ell_z} & 0 & 0 & 0 & 0\\
\vdots & \ddots & \vdots & \vdots & \vdots\\
0 & 0 & e^{iq_z(0)\ell_z} & 0 & 0\\
\vdots & \vdots & \vdots & \ddots & \vdots\\
0 & 0 & 0 & 0 & e^{iq_z(N2\pi/d_x)\ell_z}
\end{pmatrix} \tag{S13}
$$

where ℓ_z is the distance between the gratings, matches the fields in the regions of the structure indicated as U and L in Fig.S1:

$$
\mathbf{A}_U = \chi(\ell_z)\mathbf{A}_L \qquad \mathbf{B}_U = \chi(-\ell_z)\mathbf{B}_L \tag{S14}
$$

By considering the TE polarization and imposing the Maxwell boundary conditions at $z = \ell_z/2$ and $z = L_z + \ell_z/2$ we obtain the amplitudes in the transfer matrix form:

$$
\begin{aligned}
A_U(G) &= \sum_n \left[t_2^>(G,n)\right]^{-1}\eta_n(G)\left\{A_{n2} + r_2^>(G,n)B_{n2}\right\}\\
B_U(G) &= \sum_n \left[t_2^>(G,n)\right]^{-1}\eta_n(G)\left\{r_2^>(G,n)A_{n2} + B_{n2}\right\}
\end{aligned} \tag{S15}
$$

$$
\begin{aligned}
A_3(G) &= \sum_n \left[t_2^>(G,n)\right]^{-1}\eta_n(G)\left\{A_{n2}e^{ik_nL_z} + r_2^>(G,n)B_{n2}e^{-ik_nL_z}\right\}\\
B_3(G) &= \sum_n \left[t_2^>(G,n)\right]^{-1}\eta_n(G)\left\{r_2^>(G,n)A_{n2}e^{ik_nL_z} + B_{n2}e^{-ik_nL_z}\right\}
\end{aligned} \tag{S16}
$$

where:

$$
r_2^>(G,n) = \frac{q_z(G)-k_n}{q_z(G)+k_n} \qquad t_2^>(G,n) = \frac{2q_z(G)}{q_z(G)+k_n} \tag{S17}
$$

are the interface reflection and transmission amplitudes of the $j = 2$ grating layer.

It is, however, well known that[1–3] the inevitable existence of evanescent solutions in dielectric gratings makes the transfer-matrix calculation numerically unstable very quickly, so we rewrite the system in Eq.S18 in the scattering matrix form as:

$$\begin{array}{l} A_U(G) = \sum_n \left[t_2^{>}(G,n)\right]^{-1} \eta_n(G) \left\{A_{n2} + r_2^{>}(G,n)B_{n2}\right\} \\ B_3(G) = \sum_n \left[t_2^{>}(G,n)\right]^{-1} \eta_n(G) \left\{r_2^{>}(G,n)A_{n2}e^{ik_nL_z} + B_{n2}e^{-ik_nL_z}\right\} \end{array} \tag{S18}$$

$$\begin{array}{l} A_3(G) = \sum_n \left[t_2^{>}(G,n)\right]^{-1} \eta_n(G) \left\{A_{n2}e^{ik_nL_z} + r_2^{>}(G,n)B_{n2}e^{-ik_nL_z}\right\} \\ B_U(G) = \sum_n \left[t_2^{>}(G,n)\right]^{-1} \eta_n(G) \left\{r_2^{>}(G,n)A_{n2} + B_{n2}\right\} \end{array} \tag{S19}$$

for the input $(A_U(G), B_3(G))$ and output $(A_3(G), B_U(G))$ field amplitudes of the $j = 2$ grating layer.

The forward optical response of the dielectric grating layer, for a given incident in-plane wave vector $q_p(G')$, by the assumption $A_U(G) \to \delta_{G,G'}$ and $B_3(G) \to 0$ makes the system of Eq.S18 a heterogeneous algebraic system that can be solved with respect to the values of the internal electric field amplitudes A_{n2}, B_{n2}. Then the system of Eq.S19 gives the matrices of forward reflection $B_U(G) \to r_2(G,G')$ and transmission $A_3(G) \to t_2(G,G')$.

Due to the optical symmetry of the grating layer, the backward optical response gives the same reflection and transmission matrices. We can then write the scattering matrix of the system as:

$$\begin{pmatrix} \mathbf{A}_3 \\ \mathbf{B}_U \end{pmatrix} = S_2(\omega) \begin{pmatrix} \mathbf{A}_U \\ \mathbf{B}_3 \end{pmatrix} = \begin{pmatrix} t_2(\omega) & r_2(\omega) \\ r_2(\omega) & t_2(\omega) \end{pmatrix} \begin{pmatrix} \mathbf{A}_U \\ \mathbf{B}_3 \end{pmatrix} \tag{S20}$$

Analogous relations can be obtained for the first grating layer:

$$\begin{pmatrix} \mathbf{A}_L \\ \mathbf{B}_o \end{pmatrix} = S_1(\omega) \begin{pmatrix} \mathbf{A}_o \\ \mathbf{B}_L \end{pmatrix} = \begin{pmatrix} t_1(\omega) & r_1(\omega) \\ r_1(\omega) & t_1(\omega) \end{pmatrix} \begin{pmatrix} \mathbf{A}_o \\ \mathbf{B}_L \end{pmatrix} \tag{S21}$$

Moreover, Eq.S14 now is:

$$\begin{pmatrix} \mathbf{A}_U \\ \mathbf{B}_U \end{pmatrix} = \begin{pmatrix} \chi(\ell_z) & 0 \\ 0 & \chi(-\ell_z) \end{pmatrix} \begin{pmatrix} \mathbf{A}_L \\ \mathbf{B}_L \end{pmatrix} \tag{S22}$$

where the field amplitudes are given in the $\{G, G'\}$ reciprocal space, and $\chi(\pm\ell_z)$ are diagonal matrices. From Eqs.S20, S21 and S25 we obtain:

$$\begin{array}{l} \mathbf{A}_L = \mathbf{G}^{>}(\ell_z)\left[\mathbf{t}_1(\omega)\mathbf{A}_o + \mathbf{r}_1(\omega)\chi(\ell_z)\mathbf{t}_2(\omega)\mathbf{B}_3\right] \\ \mathbf{B}_L = \mathbf{G}^{<}(\ell_z)\left[\mathbf{r}_{12}(\omega)\mathbf{t}_1\mathbf{A}_o + \chi(\ell_z)\mathbf{t}_2(\omega)\mathbf{B}_3\right] \end{array} \tag{S23}$$

where:

$$\begin{array}{l} \mathbf{G}^{>}(\ell_z) = \left[\mathbf{I} - \mathbf{r}_1(\omega)\mathbf{r}_{12}(\omega)\right]^{-1} \\ \mathbf{G}^{<}(\ell_z) = \left[\mathbf{I} - \mathbf{r}_{12}(\omega)\mathbf{r}_1(\omega)\right]^{-1} \end{array} \tag{S24}$$

with: $\mathbf{r}_{12}(\omega) = \chi(\ell_z)\mathbf{r}_2(\omega)\chi(\ell_z)$

The total scattering matrix of the cavity is then:

$$\begin{pmatrix} \mathbf{A}_3 \\ \mathbf{B}_o \end{pmatrix} = \begin{pmatrix} t_2(\omega)\chi(\ell_z) & 0 \\ 0 & t_1(\omega) \end{pmatrix} \begin{pmatrix} \mathbf{A}_L \\ \mathbf{B}_L \end{pmatrix} + \begin{pmatrix} 0 & r_2(\omega) \\ r_2(\omega) & 0 \end{pmatrix} \begin{pmatrix} \mathbf{A}_o \\ \mathbf{B}_3 \end{pmatrix} \tag{S25}$$

The whole procedure can be iterated when additional grating layers are stacked to obtain the multilayer structure.

3 ABS optical parameters.

The same experimental set-up used to measure the spectral and dynamical properties of THz pulses transmitted through the photonic crystals was also used to measure the optical properties of the ABS material as a function of frequency. To this purpose transmittance of 3D printed unstructured ABS slabs with thickness of 0.5 and 2 mm were measured. The optical parameters were obtained from the measured spectra by using a numerical procedure able to remove the residual Fabry-Perot oscillations present in the spectral behaviors of samples with flat and parallel surface[4]. Results for refractive index, absorption coefficient and dielectric functions are shown in Fig. S2 in the frequency range 0.1-0.6 THz. These values are in agreement with previous measurements of ABS optical properties[5].

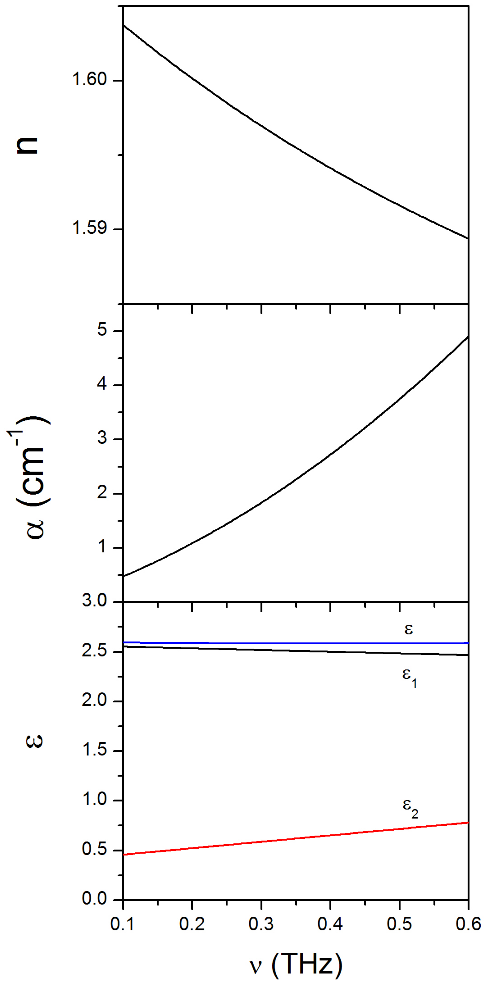

Figure S2. Optical parameters of ABS used to build the photonic structures as a function of the frequency (ν): refractive index (top), absorption coefficient (middle) and dielectric functions (bottom).

4 THz deconvolution

The deconvolution technique was applied to the THz time-domain data both for the ABS slab with a thickness of about 0.41 mm and for the photonic structures made of different layers of grids. In order to recover the impulse response function IRF(t), as a function of the time t, we follow the procedure described in Ref. 6. Results are shown in Fig. S3 and Fig. S4. As a function of the number of layers, the number of peaks in the IRF(t) and their time delay increases (Fig. S4). In addition, an overall increasing of the peaks' width is observed. Since the peaks are overall superimposed on each other, we estimated the width of the first peak appearing from 0.83 ps (1 layer) to 2.3 ps (12 layers), which is quite well separated from other peaks. The results were normalized to the width of the IRF(t) of the THz pulses transmitted in the ABS slab, which reasonably is the width of the incident THz signal. Results are shown in Fig. S5. The behaviour of the normalized pulse width can be well fitted by a linear function $1+0.186n_\ell$, very similar to that obtained by using the squared module of transmitted THz pulses $(1+0.193n_\ell)$.

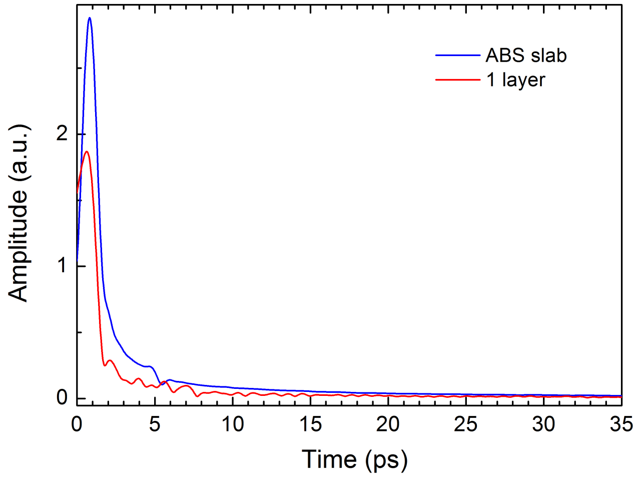

Figure S3. IRF(t) for the ABS slab and 1 layer of rods.

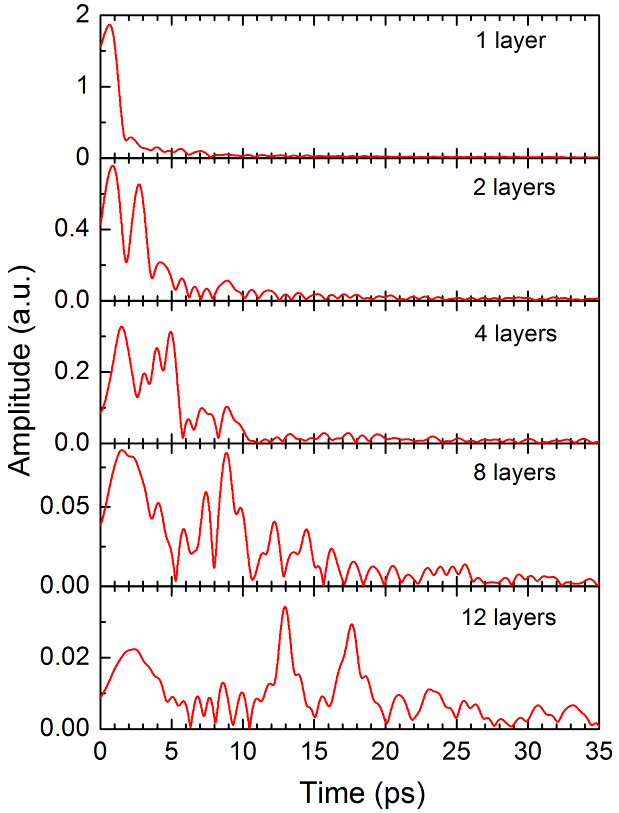

Figure S4. IRF(t) as a function of the number of layers.

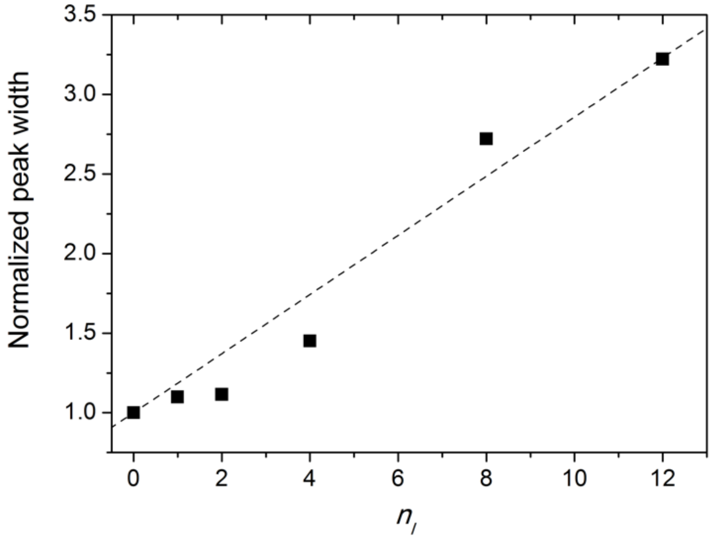

Figure S5. Normalized peak width as a function of the number of layers.

References

1. S. G. Tikhodeev, A. L. Yablonskii, E. A. Muljarov, N. A. Gippius, and Teruya Ishihara, Phys. Rev. B **66**, 045102 (2002)
2. David Yuk Kei Ko and J. C. Inkson, Phys. Rev. B **38**, 9945 (1988).
3. D. M. Whittaker and I. S. Culshaw, Phys. Rev. B **60**, 2610 (1999).
4. R. Fastampa, L. Pilozzi, M. Missori, Phys. Rev. A **95**, 063831 (2017).
5. E. Castro-Camus, M. Koch, A. I. Hernandez-Serrano, J. Appl. Phys. **127**, 21, 210901 (2020)
6. Gillian C. Walker John W. Bowen Julien Labaune J-Bianca Jackson Sillas Hadjiloucas John Roberts Gerard Mourou Michel Menu, Optics Express **20**, 25, 27230 (2012)
